# Eco-Evo-Devo of petal pigmentation patterning

**DOI:** 10.1042/EBC20220051

**Published:** 2022-12-08

**Authors:** Alice L.M Fairnie, May T.S. Yeo, Stefano Gatti, Emily Chan, Valentina Travaglia, Joseph F. Walker, Edwige Moyroud

**Affiliations:** 1 The Sainsbury Laboratory, University of Cambridge, Bateman Street, Cambridge CB2 1LR, U.K.; 2 Department of Genetics, Downing Site, University of Cambridge, Cambridge CB2 3EJ, U.K.

**Keywords:** development, evolution, patterning, Petal, pigments, pollination

## Abstract

Colourful spots, stripes and rings decorate the corolla of most flowering plants and fulfil important biotic and abiotic functions. Spatial differences in the pigmentation of epidermal cells can create these patterns. The last few years have yielded new data that have started to illuminate the mechanisms controlling the function, formation and evolution of petal patterns. These advances have broad impacts beyond the immediate field as pigmentation patterns are wonderful systems to explore multiscale biological problems: from understanding how cells make decisions at the microscale to examining the roots of biodiversity at the macroscale. These new results also reveal there is more to petal patterning than meets the eye, opening up a brand new area of investigation. In this mini-review, we summarise our current knowledge on the Eco-Evo-Devo of petal pigmentation patterns and discuss some of the most exciting yet unanswered questions that represent avenues for future research.

## Introduction

**Figure 1:**
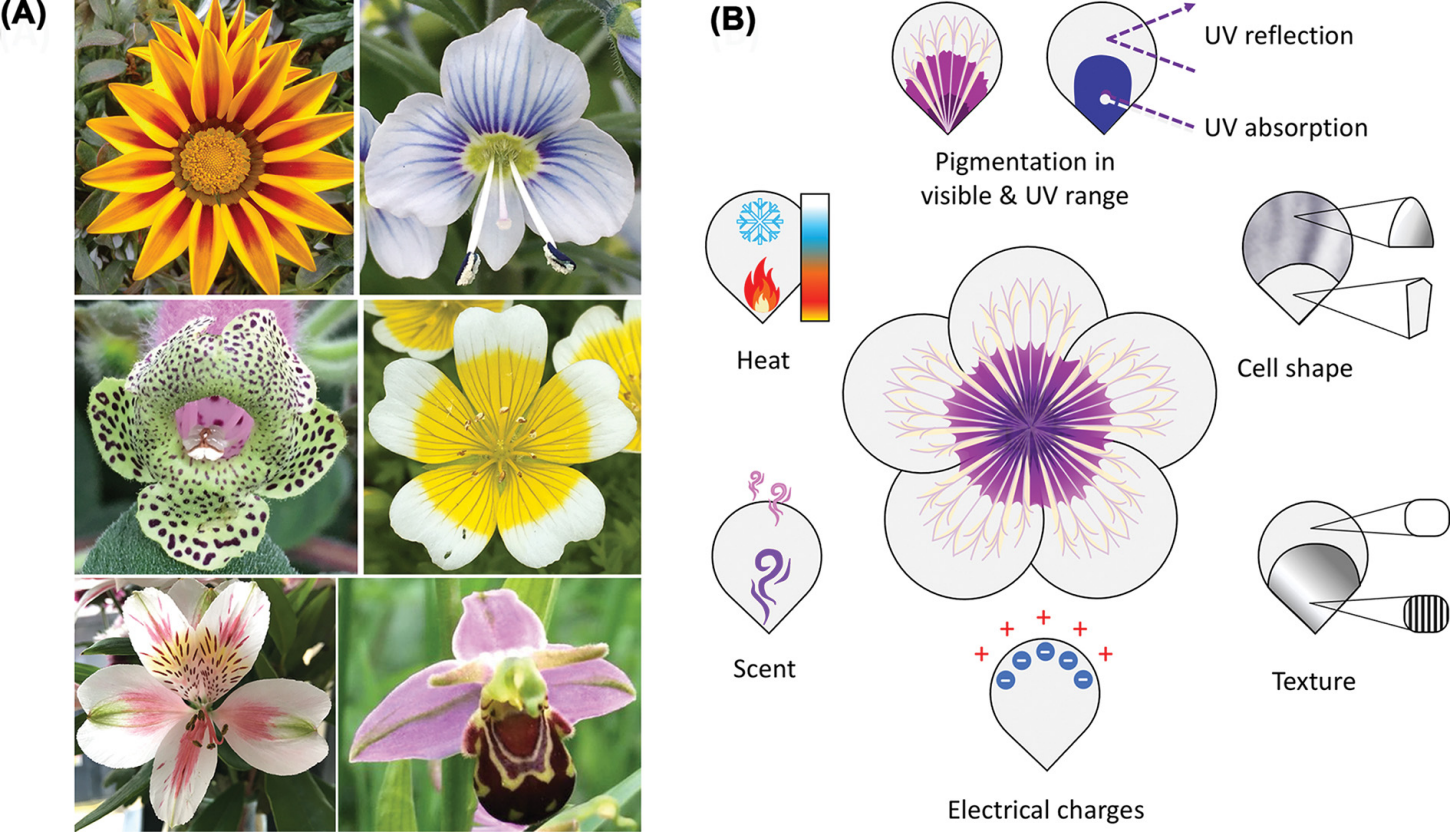
Diversity of petal pigmentation patterns and co-occurrence with other patterned cues (**A**) Examples illustrating the diversity of pigmentation patterning on the corolla of flowering plants. Top left: stripe pattern of *Gazania* sp.; Top right: venation pattern of *Veronica* sp.; Middle left: spots pattern of *Kohleria warszewiczii*; Middle right: bullseye pattern of the poached egg plant (*Limnanthes douglasii*); Bottom left: complex pattern combining spots, stripes and brushed marks in Peruvian lily (*Alstroemeria* sp.); Bottom right: Bee orchid (*Ophrys apifera*) flower displaying a pigmentation pattern participating in mimicry (female body of pollinator) and/or warning signal against herbivores. (**B**) Chemical pigments acting in the visible and UV range coexist with physical tridimensional features (cell shape and texture of the cuticle) that sculpt the petal surface. Heat and humidity gradients, electrical charges and scent emission patterns have also been reported, adding another layer of complexity to petal patterning.

Petals show striking morphological diversity across the ∼350,000 species of flowering plants, sometimes even within the same flower. When asked to describe flowers, it is often easiest to detail the differences in their petals. Patterns emerge on the petal surface when different regions of the epidermis develop distinct characteristics. Most petals are not uniformly coloured; some patterns are hidden from us and only detected by animals. Pollinating insects can perceive differences in colour, shape, scent and texture, or a combination of these as well as heat, humidity or even electrical charge gradients on the petal surface [1–9]. Pattern elements acting in combination can create complex petal surfaces, but to date, most studies have focused on a single pattern element. The best researched petal patterns are colour patterns, including patterns in the UV range of the spectrum not visible to the human eye [5,10–15]. These patterns include spots and arrows, stripes, venations, bullseyes, picotees or complex forms combining simple motifs (Figure 1).

Spots are formed by restricting pigmentation to small dots or circles of cells on the epidermis. Petal spots can vary in number, position, size or shape [13,16–18]. The shape of white spots in *Lapeirousia oreogena* is highly modified, with pointed tips resembling an arrow [19]. The pigmented midrib of Asiatic lilies tepals (equivalent to petals) constitute colourful stripes [17], but striped patterns can also be created by the absence of pigment [20–22]. As seen in snapdragons [23] and moth orchids [24], venations resemble stripes, but these pigmentation bands overlay and follow the vein patterns within the petal. Bullseye patterns result from contrasting rings of colour. The *Hibiscus trionum* bicolour bullseye is visible to the human eye, with dark anthocyanin pigmentation at the petal base contrasting with cream-coloured flavonols at the tip [25]. Sunflowers appear uniformly yellow to humans, but to pollinators, their flowers feature a bullseye due to the UV-absorbing nature of the ligule (a petal-like structure) base [15]. In extreme cases, only the outer petal margin contrasts with the centre, forming picotee patterns frequent in petunia cultivars [26]. By combining simple pattern elements, intricate motifs emerge, such as those found on the Peruvian lily corolla or the labellum of bee orchids (Figure 1).

Plant pigments belong to three main categories: flavonoids (red to blue anthocyanins and cream-yellow flavonols), carotenoids and betalains. All three groups participate in petal patterning and are highly diverse with regard to their chemical structures and the colours they produce. The properties of these pigments, along with their synthesis and their physiological and ecological roles, have been extensively studied and recently reviewed [27–33]. Patterns emerge when pigment production only occurs in designated epidermal cells or when distinct regions of the petal synthesise different amounts of a given pigment or different pigment mixtures. This process relies on restricting the expression of structural genes (i.e., enzymes from the biosynthetic pathways) or the activity of corresponding proteins to specific regions of the petal.

Transcriptional regulators of the flavonoid pathway are well-known: biosynthetic gene expression is mainly controlled by MBW complexes consisting of R2R3-MYB, basic-helix-loop-helix (bHLH) and WD-repeat (WDR) proteins, reviewed in [34]. MYB proteins serve dual roles as transcriptional activators and repressors of the biosynthetic pathway. MYB activators of anthocyanin biosynthesis all belong to one of three subgroups 5, 6 or 27, while most subgroup 4 members repress compound synthesis [34]. Our understanding of floral regulation of carotenoids and betalain production lags, but recent studies revealed that MYBs also take centre stage. The R2R3-MYB genes *WHITE PETAL1* and *Reduced Carotenoid Pigmentation 1* can regulate carotenoid-derived petal colour in *Medicago* and *Mimulus lewisii*, respectively [35,36]. MYB transcription factors (TFs) also control the synthesis of betalains but underwent changes to specialise in betalain production [37]: the mode of action of *BvMYB1*, a highly expressed subgroup 6 R2R3-MYB identified in beetroot, is unclear but it fails to interact with known bHLH partners derived from anthocyanic model organisms [37].

Petal patterns have long been a source of fascination for scientists from various disciplines. This may be why the functional relevance of those motifs, the molecular and cellular events orchestrating their formation and the evolutionary processes that modify them have often been studied independently from each other. However, the field has gained incredible momentum in the last 15 years. This is in part due to the rapid and widespread adoption of novel technologies but also thanks to powerful model systems well-suited for functional investigations at the bench as well as behavioural studies in the field. Petal patterns are more complex than they may appear to the naked human eye. By taking a multiscale and interdisciplinary approach, it is now possible to start understanding the wealth of fascinating biology, until very recently largely unexplorable, that underlies those multifunctional features. In this review, we attempt to synthesise our current understanding of the roles, development and evolution of the motifs that decorate petal surfaces. We also identify current bottlenecks and gaps in our knowledge, and we propose future strands of research to move the field forward. Finally, we discuss how recent findings position petal patterns as excellent systems to ask essential questions that reach far beyond the biology of petal patterning, allowing scientists to directly explore the inner workings of cells and the basis of biodiversity.

## Functional relevance of petal patterning

### Pollination

Petal patterns are important in pollinator interactions [13,38–41]. Colour patterns can function as communication tools between flowering plants and their visitors before or after the animal has landed, and the response of a given pollinator to a specific pattern can be innate or acquired through learning. Pre-landing signals attract pollinators to a flower while post-landing signals influence the way pollinators handle the flower. These floral guides direct pollinators towards rewarding or reproductive flower parts and can even deter robbing behaviour [42].

Pattern-mediated pollinator attraction is well commented on in the literature, but until recently, experimental validation remained scarce. In the 1990s, Dafni and colleagues demonstrated that flowers with a dark centre or with bilaterally symmetric patterns are more attractive to beetles than flowers without a dark centre, or with radially symmetric patterns [43,44]. We now know that different species or even individuals can respond differently to a given pattern: hawkmoths and swallowtail butterflies are attracted to *Hemerocallis* flowers but for different reasons. Swallowtails prefer *H. fulva* because of colour contrast within the flower. Hawkmoths prefer *H. citrina* flowers because of their UV-reflecting bullseye pattern [45]. Yellow monkeyflowers with a red patch across the nectar guide region appear more appealing to bumblebees than those producing numerous smaller spots [46]. In contrast, a UV bullseye in yellow silverweed makes flowers more attractive to bees and syrphid flies [13]. As different animal species sometimes show contrasting preferences for different petal motifs, changes in petal patterning during evolution could in theory contribute to reproductive isolation and eventually led to speciation.

Pigmentation patterns allow flowers to resemble food-rewarding species [47] or mimic the pollinating insect’s mate. Mate mimicry has been proposed for the South African daisy, *Gorteria diffusa*, which attracts dipteran flies [48]; *Linum pubsecens*, which attracts bee flies [49] and *Romulea monadelpha*, *Hesperantha vaginata* and *Sparaxis elegans*, three species pollinated by monkey beetles [50]. The ecological function of *Gorteria* dark spots was investigated experimentally with artificial flowers: circular discs with central black spots were shown to reduce the inter-floral search time of bumblebees compared with unpatterned orange discs [40].

The prevalence of venation patterns could reflect their role as pollinator guides [51], but observational tests of their exact contribution to plant–animal interactions have been lacking. Artificial flowers with radiating UV blue lines on a circular blue background or radiating yellow lines on white circular backgrounds reduce bumblebees' post-landing nectar discovery time [42,52]. *Guideless* mutants in *Mimulus* lack yellow floral guides. Not only are fewer pollinator visits recorded for *guideless*, but the incorrect entry position of visitors likely reduces pollen transfer to the insect’s body [53]. Experimental manipulations of the *Hypoxis camerooniana* pattern alter post-landing pollinator behaviour [54], demonstrating that UV bullseyes can also influence plant–pollinator interactions post-landing.

To date, only a handful of studies have specifically examined how differential visitation associated with colour patterns, rather than with overall flower colour or other well-studied floral traits such as flower size and shape, translates into individual fitness. The findings of those studies indicate that the effect of petal patterning on plants reproductive success varies broadly between species and is likely to depend heavily on the environmental context. For example, Tremblay and Ackerman [55] found that bicoloured flowers are more common than uniformly pigmented ones in various populations of a small Puerto Rican orchid. However, the authors could not detect any association between the presence of a pattern and a positive effect on male or female fitness (i.e., the ability to export pollen or the capacity to receive pollen and produce seeds, respectively) [55]. In contrast, Hansen and colleagues [19] found that removing the arrow-shaped nectar guide from the corolla of the South African iris is sufficient to almost completely suppress pollen export and to significantly reduce the ability of the plant to set fruits. In this case both male and female fitness are severely affected when a specific element of the petal pattern disappears. Importantly, the ability of petal patterns to impact on reproductive success is likely to be context dependant: in silverweed larger UV-absorbing bullseyes received twice as many foraging visitors than smaller bullseyes but this difference was only observed in individuals growing at high altitude [56]. Petal patterns that increase the frequency of foraging visits can potentially affect both male and female fitness as the chance to export and receive pollen is increased. Thus, a selection acting on pattern dimensions could act via both male and female functions but whether this is the case may depend on other factors (i.e., pattern visibility varying as light conditions change with altitude, presence of different plant neighbours).

Recent research suggests that coloured petal patterns could also fulfil functions unrelated to pollination, including response to environmental factors or interactions with pests and herbivores. These alternative functions are perhaps better thought of as additional functions because they may act alongside pollinator interactions.

### Predation

Colourful displays usually produced by flowers are occasionally found on other plant organs: various carnivorous plants display patterns within the UV light range on their trap [57], a modified leaf dedicated to prey capture. Artificial plants with UV patterns mimicking those found on the rim of pitcher plants capture more flies with normal vision than flies with reduced vision, suggesting that pigmentation patterns can serve as visual cues capable of luring prey [58]. Whether this mechanism is used by other species of carnivorous plants to attract insects remains to be established [59]. Predatory animals can even exploit pollinators’ preferences for certain pigmentation patterns: the UV-reflective body of the spider crab creates a contrasting pattern on the otherwise uniformly white corolla of daisies. The presence of the spider crab enhances the flower’s attractiveness to pattern-sensitive honeybees, which could constitute an effective tactic to ambush prey [60].

### Defence

**Figure 2:**
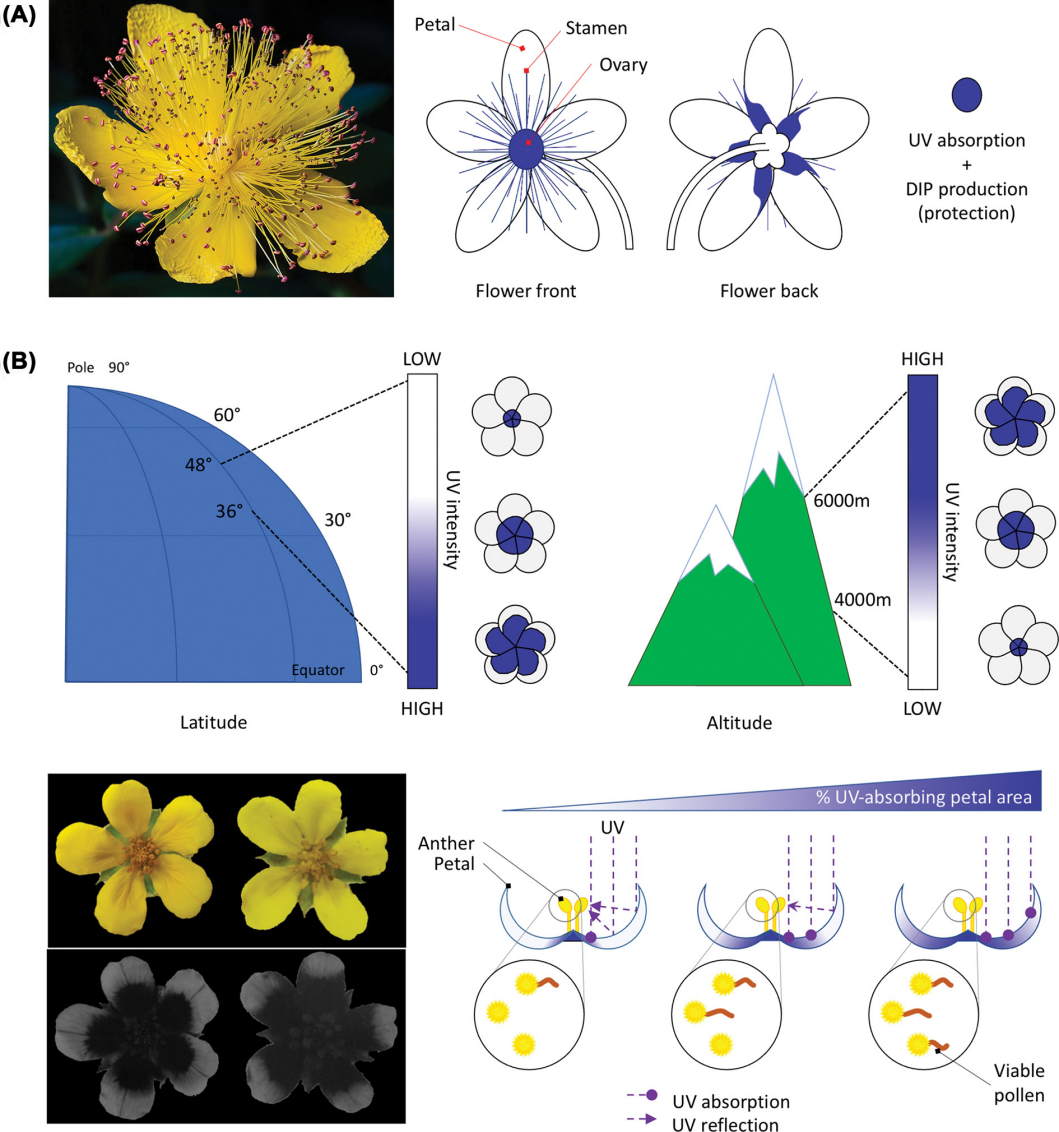
Pigmentation patterns can provide defence against herbivores and environmental conditions (**A**) The flower of St John’s wort (*Hypericum calycinum*) produces a UV bullseye that could participate in pollinator attraction (UV absorption) and provide chemical defence (DIP production) against the caterpillar herbivore of the Bella moth. (**B**) The flowers of silverweed cinquefoil (*Argentina anserina*) appear yellow to the human eye but produce a UV bullseye. The size of the UV absorbing region can vary between populations: individuals growing at lower latitudes or higher altitudes tend to form larger bullseyes. If big enough, the bullseye can effectively shield pollen grains against damage caused by UV irradiance: incident UV rays are absorbed by the bullseye centre instead of being reflected on the pollen-producing anthers in the centre of the flower. The percentage of viable pollen grains following UV treatment was found to increase with bullseye size, supporting a protective role for petal pigmentation patterns. Image credit in (A) JJ Harrison via Wikimedia Commons and in (B) Dr Matthew H. Koski.

St. John’s wort flowers exhibit a UV pattern on the back of their petals (Figure 2A). This pattern bears a mixture of de-aromatised isoprenylated phloroglucinols, including hypercalin A, a deterrent toxic to the caterpillar herbivore of the Bella moth [61]. The UV-absorbing compounds could appeal visually to pollinators by creating a bullseye involving the flower’s reproductive organs and providing protection against herbivores to pollen and seeds. Animals commonly use colouration as a strategy to avoid predation. Predation-avoidance strategies also occur in plants, in particular as defence against herbivores [62,63]. A role for petal colour pattern as a warning signal has been proposed but needs to be fully tested. Bee orchids (Figure 1) may attract pollinators through scent mimicry alone as the visual details on the flower seem too delicate for bees which have limited spatial resolution. In this instance, the visual pattern on the corolla may instead deter herbivorous mammals [64]. Ants are known to protect certain species of plants from herbivores and nectar thieves, and black markings on the petals of several species of passion fruit flowers could mimic those beneficial insects [65,66].

### Abiotic functions

Petal pigmentation patterns can also fulfil abiotic functions: the size of the UV-absorbing bullseye of silverweed co-varies with UV incidence as populations experiencing higher UV irradiation display a larger UV-absorbing centre [12] capable of shielding pollen grains from damaging UV rays more efficiently than a smaller UV-absorbing bullseye (Figure 2B). Local pigment accumulation, especially UV-absorbing compounds, can also generate temperature and humidity gradients on the petal surface by capturing heat from the sun or by reducing water loss [14,67,68]. Patterned species across the Potentilleae tribe experiencing lower temperatures and sunflower populations growing in drier and colder sites tend to produce larger UV-absorbing bullseyes [15,69]. These observations support the idea that pattern dimensions may be adaptive, limiting cooling via transpiration and preventing drought stress. Because bumblebees can perceive temperature and humidity gradients [1–3], this raises the interesting possibility that pigmentation patterns directly influence the patterning of other cues.

## Developmental processes governing petal pattern formation

Variegation, colour flecks and stripes can occur due to viral infections triggering ‘colour breaks’ and genetic instability caused by transposon activity [10,70,71]. However, pattern formation is usually tightly regulated in place and time. Thus, petals are emerging models for understanding two intimately linked developmental processes: (i) patterning, which creates regions of distinct cell types, and (ii) growth, which increases tissue size, generates organ shape and acts as a modifier of patterning processes [72,73].

During petal development, the activities of the transcription factors that regulate pigmentation pathways, cell fate specification and tissue growth are likely to be set by pre-patterns [10]. These pre-patterns, set by region-specific expression of upstream genes prior to tissue differentiation, define spatiotemporal boundaries and subdomains within the epidermis. While mechanisms that could provide this spatial information have been theorised extensively, the true regulatory mechanisms have remained, until recently, largely unexplored in petals.

### Overlapping expression domains

**Figure 3:**
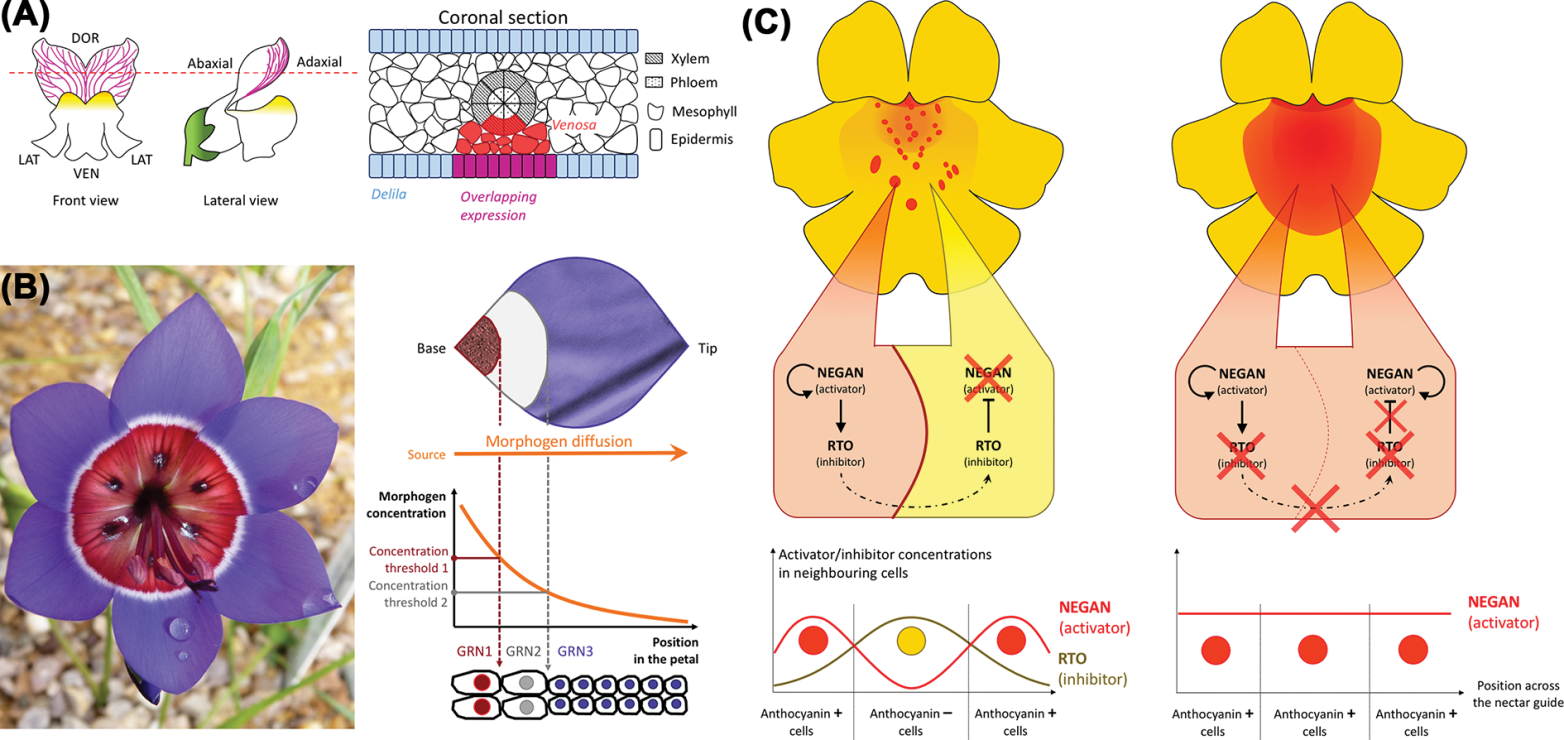
Positional and self-organisation processes regulating pigmentation pattern formation The formation of pigmentation patterns on the petal epidermis can be orchestrated by positional mechanisms (A,B) and self-organising processes (C). (**A**) The venation pattern in a subspecies of snapdragon is specified by overlapping expression domains of a MYB transcription factor, *VENOSA*, and its bHLH partner, *DELILA*. As both components need to be present for the MBW complex to function, anthocyanin production is only triggered in the few epidermis cells situated just above the petal vasculature. (**B**) Morphogen diffusion could in theory pre-pattern the epidermis of the developing petal by forming concentration gradients along their proximo-distal axis. Different morphogen concentrations could activate distinct genetic programs to specify multiple cell fates and the production of various pigment mixtures in different portions of the petal. Such a mechanism could account for the production of the striking bullseye of the South African *Geissorhiza radians*. (**C**) Left: formation of red anthocyanin spots on the corolla of monkeyflowers is regulated by a reaction-diffusion system involving an activator of pigment synthesis, NEGAN, and its diffusing repressor, RED TONGUE (RTO). Right: in *rto*/*rto* homozygous mutants, the lack of inhibitor function yields flowers with a near-uniform patch of anthocyanin colour at the base of their petals.

Expression of each component of the MBW complex is critical as the complex is only active when all components are present. This provides grounds for emergence of precise pigmentation patterns from broadly defined expression domains. In snapdragon, venation patterning requires the R2R3 MYB *VENOSA* and its bHLH partner DELILA. *VENOSA* is expressed around the vasculature while *DELILA* transcription is restricted to the petal epidermis, defining a narrow overlapping domain in the epidermal cells overlying the vasculature where partners coexist and activate anthocyanin production [23] (Figure 3A). However, it is unclear what sets the expression domains of MYB and bHLH components in the first place. For certain types of patterns, the required positional information could be specified through signalling from an existing differentiated region. Here, the vasculature may provide the localised signal to activate *VENOSA* expression. This signal could be auxin as *VENOSA* has two auxin-response element-like sequences in its promoter [23].

### Morphogen gradients

Morphogens can also provide positional information (Figure 3B). This notion is well defined in animals where growth and patterning can be coordinated by diffusible signals produced at a localised source and forming concentration gradients across a tissue. These gradients modulate cell fate and proliferation in a concentration-dependent manner to convey positional information [74]. The existence of morphogens in plants is still under scrutiny [75], although auxin, small RNAs, peptides or even transcription factors could fulfil a morphogen-like role [76–79]. In roots, the combined concentration gradients of auxin and PLETHORA (PLT) transcription factors spatially organise three distinct zones where cells proliferate, elongate and finally differentiate in a threshold-dependent manner [80]. In petals, *PLETHORA3* also regulates cell proliferation and differentiation in a dose-dependent manner [81]. However, whether a PLT3 concentration gradient exists in developing petals is unclear. Opposite gradients of the small RNA miR396 and the GIF1/AN3 TF shape *GROWTH REGULATORY FACTOR (GRF)* activity along the proximo-distal axis of developing leaves [82]. The *gif1*/*an3* mutation also affects petals, yielding shorter, narrower petals [83,84]. However, the expression and activity domains of miR396, GRFs and GIFs remain to be characterised with spatiotemporal resolution in floral organs to determine whether the miR396-GRF/GIF module can pattern petal cell proliferation and differentiation.

### Self-organisation

Spontaneous acquisition of positional characteristics without apparent pre-patterns is common in animals and plants [10,75,85,86]. Self-organising pattern formation is often explained by reaction-diffusion models. These models postulate that patterns arise through instabilities generated by the interplay between at least two molecular factors, namely an activator which activates its own production, and an inhibitor that represses the activator [87,88].

The development of red spots in *Mimulus guttatus* is the product of a two-component reaction-diffusion system involving an R2R3-MYB activator, NECTAR GUIDE ANTHOCYANIN (NEGAN), and the R3-MYB diffusible inhibitor, RED TONGUE (RTO) (Figure 3C). NEGAN activates anthocyanin production at the petal base. NEGAN also promotes its own transcription along with the expression of *RTO*, which acts as a long-range inhibitor of NEGAN to regulate spot formation [46]. Anthocyanin pigmentation does not extend across the entire petal epidermis in the *rto* mutant but is restricted to the proximal half, producing a ‘red tongue’ (Figure 3C). This phenotype reveals the existence of positional information, acting upstream of the reaction-diffusion mechanism, to restrict the initial expression of *NEGAN* to the proximal region. Thus, monkeyflower petals utilise pre-patterning processes (that set the initial conditions and restrict pattern formation in space) and self-organising mechanisms (that act downstream and specify the type of pattern) to add spots to the petal base.

Modelling approaches are powerful ways to explore pattern emergence, especially as the processes involved are often not intuitive. By combining mathematical models of relatively simple mechanisms, Ringham and colleagues recapitulated a broad range of petal patterns observed across flowers [89]. A theoretical model indicates that an activator-repressor system could also account for the violet and white pigmentation rings characteristic of passion flowers, although the molecular players remain to be identified [90]. A modified form of the reaction-diffusion system, the substrate-depletion model, has been proposed to explain haloed spot formation in foxgloves, where petal spots are surrounded by a zone of reduced pigmentation [10]. Models for spot-halo formation are based either on the export of R3-MYB repressors from the spot cells or depletion of an activator or biosynthetic substrate (e.g., WDR co-regulators) from cells surrounding the spot. However, whether a substrate-depletion mechanism can account for spot-halo formation remains to be demonstrated.

### Contribution of structural elements

Pigment appearance can change according to their subcellular location and the shape of the cell. Cell shape and cuticle texture constitute tridimensional structures that impact the path of light entering or exiting the cell, modifying the visual aspect of the petal or even producing colours through light diffraction and constructive interference [4,91–97]. Thus, colour patterns could, in theory, emerge not by modifying pigment production locally but by varying cell shape and texture across the petal surface (Figure 1B). Much remains to be understood about the mechanisms controlling cell shape geometry and nanopatterning of the cell surface [92]. Whether and how the processes regulating the distribution of physical features or the production of chemical compounds across the petal epidermis are coordinated is unknown and represents an exciting strand of investigation.

### Mechanisms of petal pattern evolution

**Figure 4:**
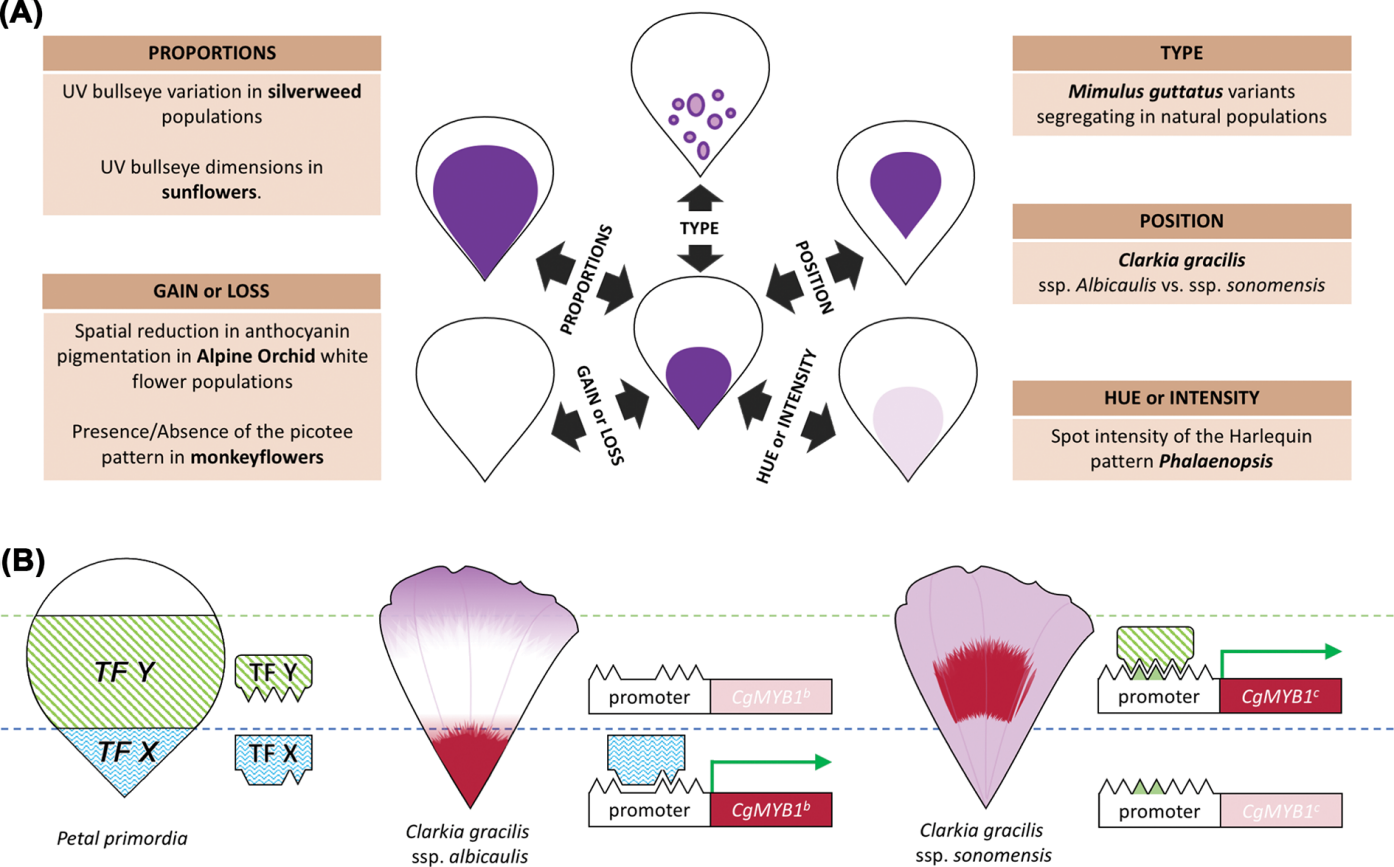
Mechanisms associated with pigmentation pattern evolution (**A**) Possible modifications affecting petal pigmentation patterns (centre) and examples from the literature corresponding to each case. (**B**) A shift in petal spot position in *Clarkia gracilis* occurred following mutation(s) in the regulatory region of *CgMYB1*: expression of an ancestral *CgMYB1^b^* allele is thought to have been activated by an unknown TF, TF X, restricted to the petal base. This results in the production of basal petal red spots similar to the one of subspecies *albicaulis*. Mutations in the promoter of *CgMYB1* would have resulted in the loss of TF X *cis*-elements, but a gain of binding sites for another unidentified activator, TF Y, expressed in the middle of the petal. The new allele *CgMYB1^c^* is capable of triggering spot production higher up along the proximo-distal axis of the *sonomensis* subspecies.

Petal pigmentation patterns can be gained, lost or experience changes in colouration hue or intensity during evolution. The relative position of pattern elements or their proportions can be altered, and pigmentation patterns can convert from one type into another (Figure 4A). By investigating pattern variations between species, varieties or even populations, we are beginning to identify the molecular and cellular processes that mediate pattern modifications.

Shifts in colour patterning appear predominantly linked to modified expression patterns of *R2R3-MYB* regulatory genes. *Mimulus lewisii* flowers display a white ring around their corolla throat, but the resulting picotee pattern is absent in *M. cardinalis*. Such difference between closely related monkeyflowers is due to *cis*-regulatory variation that triggers the expression of *LIGHT AREAS1*, an R2R3-MYB that promotes flavonol production, around the throat margin of *M. lewisii* [16].

Members of the *Clarkia gracilis* species complex can be distinguished by the position of their petal spot [18] (Figure 4B). The basal position is thought to be ancestral, and the shift likely occurred via *cis*-regulatory rewiring: the position of the spot is governed by distinct alleles of the *CgMYB1* gene, whose expression patterns match either the basal (*CgMYB1^b^*) or central (*CgMYB1^c^*) regions accumulating anthocyanins [18]. The authors postulate the existence of two upstream regulators, TF-X and TF-Y, whose expression is limited to the proximal or mid-section of the petal, respectively, in earlier stages of petal development. Changes affecting the regulatory region of an ancestral *CgMYB1^b^*-like allele led to *MYB1* losing its ability to be activated by TF-X while becoming controlled by TF-Y (Figure 4B). The identities of such pre-patterned regulators and the mechanisms accounting for their localised expression remain to be understood.

Mutations affecting gene coding regions can also trigger changes in petal patterning. The transition from pink to white in the petal base of *Clarkia gracilis* ssp. *sonomensis* is due to a truncated CgsMYB12 (1bp deletion in exon 3 leading to an early stop codon), which normally regulates anthocyanin production [98]. *M. guttatus* populations from Oregon and California show variation in patterning of their yellow flowers, ranging from having a fully red corolla throat (red-tongue bullseye) to a red spotted corolla throat. The transition from spot to bullseye across those wild populations is due to different alleles of *RTO*, with the red-tongue bullseye morph only carrying non-functional *rto* alleles [46] (Figure 3C).

Recently, gene duplication of R2R3-MYB was put forward as a facilitator of pattern diversification [99]: duplicates can retain their ability to activate pigment production but gain more restricted expression domains, providing numerous possibilities to create new pigmentation patterns – for instance, the uniform pink background pigmentation of *Clarkia gracilis* ssp. *sonomensis* petals results from the combined action of not one but two close paralogs, MYB11 and MYB12 [98]. Both TFs can induce pigment synthesis when ectopically expressed in *Arabidopsis*. *CgsMYB11*, is normally expressed across the petal epidermis, except at the very base. *CgsMYB12* by contrast is only expressed in the proximal portion of the petal. Expression patterns of these two MYBs together led to uniform anthocyanin production across the petal in the wild-type. But a ‘white cup’ phenotype is observed in individuals with a mutated *CgsMYB12* locus preventing anthocyanin synthesis in the basal region. Similar studies in different species scattered across the angiosperm phylogeny are now required to identify possible trends and to evaluate the relative contribution of gene duplication to the emergence of novel petal patterns. Contraction of the expression domain of *MYB12* and *MYB11* in other *Clarkia* subspecies has also been proposed [99], but our ability to test such hypotheses is currently limited by the lack of knowledge regarding the regulatory mechanisms acting upstream of MYB genes.

Petal pigmentation patterns also evolve through selection on small regulatory RNAs. Bradley and colleagues revealed that the *SULF* locus drives the production of a yellow highlight on the corolla of the *pseudomajus* subspecies of snapdragon, guiding pollinating bees to the flower entrance [100]. This locus is under selection and emerged from a genomic inverted duplication event. *SULF* codes for small RNAs that target the transcripts of Am4’CGT, the enzyme that produces the yellow pigment, leading to its post-transcriptional silencing. In *pseudomajus*, SULF is transcriptionally active throughout the corolla except for a small portion of the ventral petal marking the flower entrance, forming the nectar guide emblematic of this subspecies of snapdragon.

From an evolutionary viewpoint, petals can be seen as modified leaves [101] and gene regulatory networks controlling lateral organ patterning could be shared between petals and leaves. Pigmentation pattern on leaves is less conspicuous than those displayed by flowers and their function remains unclear. As such, the production of leaf pigmentation patterns has received little attention. Work in clover and *Medicago* identified R2R3-MYBs linked to different types of leaf markings [102,103]. One of those factors, RED HEART1 (RH1), interacts with bHLH and WD40 members to activate anthocyanin production [103]. This points towards a role for MBW complexes in leaf pigmentation patterning and supports the idea that floral MBW complexes could have evolved from leaf MBW complexes. It remains to be seen whether this only applies to the MBW complex or whether the upstream regulatory processes that divide the petal into sub-regions are also inherited from a leaf-like ancestor. While we know little about pigmentation pattern regulation in the leaf, the upstream processes that regulate lateral organ polarity and patterning have been extensively studied in leaves [104,105]. Future studies should test to what extent the mechanisms dividing emerging leaves and petals into subdomains are variations from a common ancestral system that creates boundaries across all flowering plant lateral organs.

Changes in petal pigmentation patterns could lead to reproductive isolation and speciation [106–108]. Studying the mechanisms leading to variation in petal motifs is therefore important to understand the sources of natural diversity. Data generated in the last few years indicate that changes in pigmentation patterns also represent adaptations to environmental factors such as geography and climate [12,69]. A study exploiting herbaria collections from North America, Europe and Australia indicated that UV-absorbing bullseyes have increased in size globally since the mid-twentieth century: in saucer-shaped flowers, the proportion of UV-absorbing pigment in the petal epidermis increased when ozone levels declined and decreased in locations where ozone levels increased. This trend identifies floral pattern variations as a rapid response of plants to anthropogenic climate changes [14]. The UV bullseye pattern size in some sunflowers is defined by roles in both pollinator attraction and desiccation resistance with medium-sized UV-absorbing centres attracting more pollinators. In contrast, larger-sized UV-absorbing centres offer better protection against desiccation [15]. These findings perfectly illustrate that overall changes in petal pigmentation patterns are likely to be under the influence of multiple, and sometimes opposing, biological and environmental factors.

## Future directions

Colourful flower patterns are major contributors to the diversity of the natural world. The last decade has greatly expanded our understanding of the development and evolution of flower patterns, but many questions remain. Functional investigations in novel model systems suggest that changes affecting MYB genes are often associated with variations in petal patterning during evolution. MYBs are well-known regulators of pigment synthesis hence family members are often selected first as candidate genes, but other family of pigment patterns regulators are likely to exist and could also contribute to the evolution of colourful motifs on the corolla of flowering plants. Several studies have shown that petal patterns can be modified when MYB coding sequence or regulatory elements are modified. Whether gene duplication followed by neo/subfunctionalisation or co-option of existing MYBs are equally likely to contribute to the emergence of new pattern elements remains to be tested. Importantly, identifying which *MYB* gene regulates pigment production in each sub-domain of the petal is important, but to understand the evolution of petal patterns it is now crucial to resolve the upstream mechanisms that regulate the spatio-temporal expression of those *MYBs*. Hence, it will be essential to uncover the identity of the pre-patterned regulators and signalling processes that partition the petal surface in early phases of primordia development and this represents an exciting venue for future evo-devo research.

The petal epidermis is a sensory billboard that combines diverse cues animals can perceive. Localised synthesis of chemical pigments is only one component of flowering plants’ palette. It is now evident that the elaboration of physical features such as cell shape and texture, scent emission and the distribution of heat and humidity can also be precisely patterned across the petal surface [2,3,7,9,92]. How these elements impact each other, and to what extent the production of these distinct features is regulated by shared or independent processes remain to be investigated.

**Figure 5:**
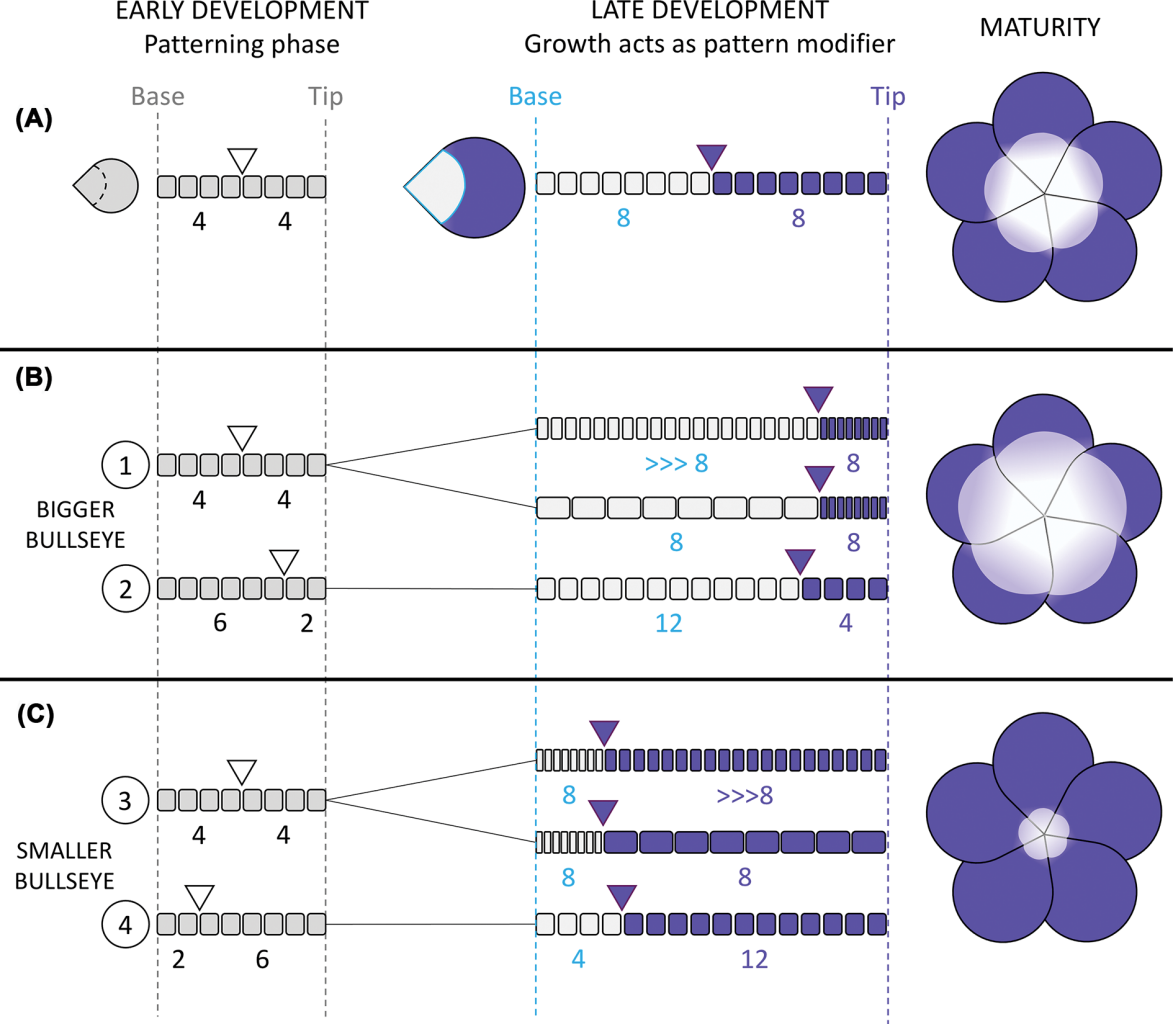
A possible role for growth as a modifier of pigmentation pattern during evolution Change in pigmentation pattern dimensions can result from modifications in early pre-patterning mechanisms that divide the petal primordia in subregions and/or local variation in cell proliferation or expansion in developmental stages following the pre-patterning phase. (**A**) Schematic representation of a theoretical petal primordium at very early stages of development, prior to pigmentation pattern formation. In this early phase, pre-patterning mechanisms act to divide the petal surface in subregions that can later develop distinct cell fates. Here proximal and distal regions are specified: the white triangle indicates boundary position along a line of 8 cells going from the base to the tip of the petal. As development progresses, cell division increases total cell number and cells acquire distinct identities in each compartment: cells in the distal region synthesises pigments while cells in the proximal domain remain unpigmented, forming a visible bullseye pattern. (**B**) Larger bullseyes can be obtained by at least two modes: in (1), the early pre-patterning processes remain unchanged and the boundary between proximal and distal regions is specified as in (A) but in later stages, an increase in either cell division rate (top lane) or cell expansion (bottom lane) in the proximal region would modify the final proportions, yielding a larger unpigmented region. Alternatively, in (2), the early boundary is specified higher along the proximo-distal axis (e.g. increase in morphogen production) so that a larger proximal compartment is specified. (**C**) Similar scenarios can lead to a reduction in bullseye size: (3) cell division (top lane) or expansion (bottom lane) can be increased in the distal region post-patterning or (4) the early developmental boundary can be specified closer to the petal base during the pre-patterning phase. White triangle: position of the early developmental boundary; purple triangle: position of the bullseye boundary in the mature petal; squares and rectangles: individual cells along the proximo-distal axis; numbers under each line of cells: cell number in each petal subregion.

Patterns displayed on mature tissues of opened flowers result from the combined effects of patterning mechanisms that specify cell fate and pattern modifiers such as growth. The roles that cell proliferation and expansion play in controlling pattern proportions and whether or not those processes can also be targeted by evolution to generate diversity remain to be understood. In Figure 5, we illustrate possible roles for growth as a modifier of coloured pattern dimensions during evolution. The development of novel model systems amenable to both lab work and field studies, along with technological breakthroughs such as single cell RNAseq and live imaging to study differentiation and growth at the cellular resolution, opens unprecedented paths to address these questions.

Although it is now clear that visiting animals can discriminate flowers based on presence/absence of petal patterns, it remains to be seen to which extent variation in pattern dimension influence pollinator behaviour before and after landing. In addition, differential visitation does not automatically translate into effects on the fitness of individuals. First, not all visitors perform a pollination services and distinguishing mere visitors from actual pollinators require careful experimental designs. Petal patterns acting as long-range signals that increase flower conspicuousness would be expected to increase male fitness (i.e., pollen export capability). Similarly, pigmentation motifs acting as post-landing guides could positively affect female fitness (i.e., pollen reception and seed production capabilities) by manipulating the position of the insect body in a way that maximise pollen deposition on stigmata, enhancing the chance of ovule fertilization and seed formation. However, the roads to male and female fitness are complexes: many external factors such as abiotic environmental conditions or the presence of different neighbour competing for animal attention as well as circumstances intrinsic to each species (e.g., the ability self-pollinate) all play a role and must be accounted for. One of the most exciting challenges for future studies will be to disentangle the contribution, if any, of petal patterns to reproductive fitness and identify precisely, in a diversity of systems, the mechanisms by which colourful motifs can influence pollination success to see if trends can be identified.

Petal patterns provide experimental and theoretical scientists with numerous opportunities for collaborative and interdisciplinary work. They also constitute excellent systems to understand how decisions made by minute cells are coordinated at the tissue and organ scale, how the execution of different developmental programs impact each other, and how evolutionary processes and diverse biotic and abiotic factors can drive and shape biodiversity. Thus, studying petal patterns help address fundamental questions of modern biology that reach far beyond understanding how flowering plants paint their surface.

## Summary

- Underlying petal pigmentation patterns is a wealth of fascinating biology that until very recently has been largely unexplorable.
- Petal patterns are multifunctional: they participate in plant–pollinator communication but also mediate interactions with herbivores, protect pollen grains from UV radiation, or enhance resistance to desiccation.
- Pigmentation motifs coexist with other patterns inconspicuous to the human eye because they are microscopic (i.e., cell shape) or because they are by nature invisible (i.e., temperature gradients).
- The petal epidermis is an excellent system to study the dynamics of cellular decision-making processes because the outcomes of decisions made by cells at the microscopic level are readily observable and can directly impact reproductive success at the macroscale.
- Petal pigmentation patterns can vary between populations and species: they can be lost or gained but also change in shape and dimension during evolution. Those changes can be adaptive with both abiotic and biotic factors able to exert selective pressures. Pattern variations can be used as markers of climate change but also lead to reproductive isolation and speciation.
- Future research will aim at understanding the mechanisms used by epidermal cells to make decisions, how these decisions are coordinated at the tissue and organ scale, what the origins of regulatory networks that govern petal patterning are and how rewiring of these networks contribute to plant fitness and the creation of biodiversity.

## Open Access

Open access for this article was enabled by the participation of University of Cambridge in an all-inclusive *Read & Publish* agreement with Portland Press and the Biochemical Society under a transformative agreement with JISC.

## Abbreviations

bHLH: basic-helix-loop-helix
MBW: MYB-bHLH-WD40 complex
TF: transcription factor
WDR: WD-repeat
